# Comparative transcriptome analysis provides insights into molecular pathway and genes associated with head-type formation and phenotypic divergence in Chinese cabbage

**DOI:** 10.3389/fgene.2023.1190752

**Published:** 2023-05-09

**Authors:** Chuan Meng, Xiaodong Liu, Fang Wu, Lei Ma, Yuhai Wang, Jingui Mu, Mingqiu Wang

**Affiliations:** Institute of Economic Crops, Hebei Academy of Agriculture and Forestry Sciences, Shijiazhuang, Hebei, China

**Keywords:** comparative-transcriptome, leafy head, Chinese cabbage, transcription factors, phytohormone-related genes

## Abstract

**Background:** The heading type of Chinese cabbage is a significant commercial trait with high economic value. At present, research on the phenotypic divergence and formation mechanism of heading type is limited.

**Results:** Through comparative-transcriptome analysis, the formation and phenotypic divergence mechanism of the leafy head of diploid overlapping type cabbage, diploid outward-curling type cabbage, tetraploid overlapping type cabbage, and tetraploid outward-curling type cabbage were systematically and comprehensively investigated, and the phenotype-specific genes of four varieties were revealed. These phenotype-specific differentially expressed genes (DEGs) were considered crucial for cabbage heading type through WGCNA. Some transcription factors have been predicted as significant genes for phenotypic divergence, including the members of the bHLH, AP2/ERF-ERF, WRKY, MYB, NAC, and C2CH2 families. Phytohormone-related genes, including abscisic acid/auxin hormone, may play an important role in the phenotypic divergence of head type in cabbage.

**Conclusion:** Comparative-transcriptome analysis supports a role for phytohormone-related genes and some transcription factors in head-type formation and divergence for four cultivars. These findings increase our understanding of the molecular basis for pattern formation and divergence of the leafy heads of Chinese cabbage and will contribute to developing more desirable leafy head patterns.

## Introduction

Chinese cabbage (*Brassica rapa ssp. pekinensis*) is one of the largest vegetable crops in planting area and market sales in Chinese vegetable production and is also the most commonly consumed vegetable in northern China (Li et al., 2022). The type of leafy head is an important agronomic trait of Chinese cabbage, which can be divided into overlapping, outward-curling, inward-curling without overlap, and spiral types (Gu. 2017). Among these, the most commonly cultivated Chinese cabbage is the overlapping type, which is also the preferred type for both growers and consumers. The overlapping type head leaves curl inward at the top with the curl length exceeding the vertical central axis of the leafy head. The overlapping presents a closed top, cleaner inner leaves, and a higher net-to-head ratio and facilitates easier packaging and transportation. In contrast, outward-curling types produce leaves that curl outward and appear open at the top (Gu. 2017). The formation of the leafy head is a complex regulatory network affected by many factors that control the earliness of the heading time in Chinese cabbage. The molecular regulatory mechanism leading to the formation and morphological disparity of Chinese cabbage leafy head is still elusive. The roles of *BrARF3.1* and *BrKAN2.1* in determining the formation of leafy heads have been researched through comparative genomic analysis (Cheng et al., 2016). Changes in concentration of the auxin (indole-3-acetic acid or IAA) between the adaxial and abaxial sides cause leaves to curl inward to form a leafy head. He et al. (2000) introduced IAA-related genes into Chinese cabbage and found that the transgenic plants showed an earlier heading, produced more leaves, and developed heavier heads. However, there is no study on the molecular mechanism that underlies the formation of the heading type in tetraploid Chinese cabbage.

Previous studies have mainly focused on the formation of the leafy head organ, its weight, height, and diameter, and the leaf bending of diploid Chinese cabbage (Kou, 2021; Wang T. et al., 2022; Yue et al., 2022a; Wang Y. et al., 2022). Genome-wide sequencing of Chinese cabbage has shown that the genes related to auxin synthesis, transport, and signal transduction pathway may be a class of important gene regulators of the morphological differences of leafy heads (Li et al., 2019; Ren et al., 2020; Yue, 2022b). Endogenous hormones in Chinese cabbage may play an important role in the formation of the leafy head: it has been reported that these participate in the development of the leafy head by regulating transcription factors, protein kinase, and calcium (He et al., 2000). Li et al. (2019) sequenced the isolated population of the diploid Chinese cabbage mutant A03 and found that the content of endogenous hormones IAA and ABA affected the heading traits of Chinese cabbage. Transcriptome analysis shows that BrAN3 plays a key role in the formation of Chinese cabbage leafy heads, and that silencing BrAN3 regulated the hormone signal pathway of auxin and ABA in Chinese cabbage (Yu et al., 2019). These findings on diploid leafy head types provide a guide for the study of the formation mechanism of tetraploid leafy head types. In recent years, tetraploid Chinese cabbage has become a research hotspot due to its increased biological yield, enhanced adaptability, disease resistance, cold tolerance, and drought resistance (Wang et al., 2018). In this study, diploid overlapping type cabbage, diploid outward-curling type cabbage, tetraploid overlapping type cabbage, and tetraploid outward-curling type cabbage were used to systematically and comprehensively explore the formation and phenotypic divergence mechanism of the leafy head. The morphological characteristics of the four cultivars were measured in detail, and flow cytometry was used to analyze the ploidy of Chinese cabbage. RNA-seq was applied to identify the pathways and genes related to these four heading type phenotypes, providing theoretical and technical support for diploid and tetraploid Chinese cabbage breeding.

## Materials and methods

### Plant materials, cultivation, and sample collection

**FIGURE 1:**
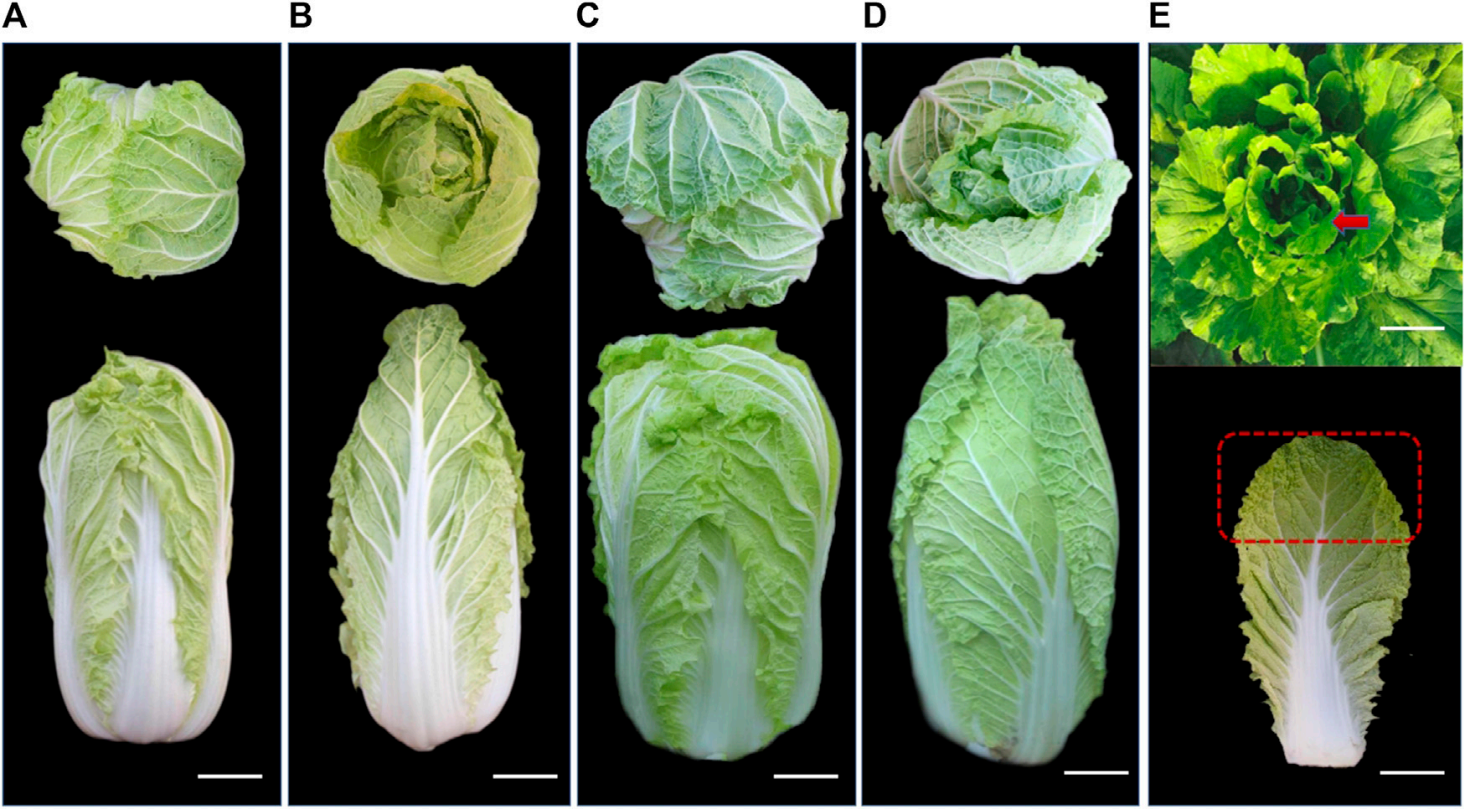
Four types of head leaf patterns in the top region for cabbage: diploid overlapping type cabbage **(A)**, diploid outward-curling type cabbage **(B)**, tetraploid overlapping type cabbage **(C)**, tetraploid outward-curling type cabbage, **(D)** and sampling site **(E)**.

Four phenotypic Chinese cabbages were used in this experimental study: diploid overlapping type cabbage, diploid outward-curling type cabbage, tetraploid overlapping type cabbage, and tetraploid outward-curling type cabbage—named D2X, S2X, D4X, and S4X, respectively. Diploid overlapping type cabbage and diploid outward-curling type cabbage were produced from diploid high-generation inbred Chinese cabbage (Figures 1A, B). Tetraploid overlapping type cabbage and tetraploid outward-curling type cabbage were produced by crossing common diploid cabbage material BP058, which can produce 2n gametes, with tetraploid cabbage materials Q29 and Q81, which were obtained by mutagenesis (Figures 1C, D). The top of the leaves (the seventh leaf from the inside to the outside) of the four cultivars were taken at the pericardium stage (56 days after planting) (Figure 1E). Three strains were selected, each of which was repeated thrice. All samples were frozen in liquid nitrogen for RNA-seq analysis and qRT-PCR expression analysis.

### Identification of ploidy by flow cytometry

To a culture dish was added 1 cm^2^ of Chinese cabbage cotyledons. The Chinese cabbage cotyledons were dissociated using 1.5 mL of extraction buffer solution and then cut with a blade. The aforementioned solution was filtered into a 2-mL centrifuge tube and centrifuged for 1000 revolutions for 3 min. After the supernatant was poured out, 50 ul of extraction buffer was added to the precipitate and shaken to suspend the precipitate. Following this, 50–100 ul of dye solution was added to the suspension and dyed for 1 min. A centrifuge tube was put into the flow cytometry (model CytoFLEX) to detect the ploidy of Chinese cabbage, and the diploid variety Chinese cabbage “Jibai 4” was used as the control. Three replicates were taken for each sample.

### RNA isolation, cDNA library preparation, transcriptome sequencing, and RNA sequencing data analysis

Total RNA from each leaf sample was isolated using TRIzol reagent (Invitrogen, Carlsbad, CA) according to the manufacturer’s instructions. The cDNA library preparation and transcriptome sequencing followed the related literature (Zhong et al., 2011; Liu et al., 2015). Clean reads were mapped to the cabbage reference genome (brassicadb.org/brad/datasets/pub/Genomes/Brassica_rapa/V3.0/) using TopHat2 software (Kim et al., 2013); only unique mapping reads were retained for calculating gene expression. RNA-seq data analysis was performed according to previously published protocols (Trapnell et al., 2010). DEGs were screened by the following standard: FDR <0.05 and absolute value of log_2_ratio ≥1. All genes were aligned against public databases (Nt, Nr, COG, Swiss-Prot, and KEGG) to obtain their putative functions. Similarly, mapping against KEGG genes involved the use of KOBAS v. 2.0 software (Xie et al., 2011) using a blast cutoff e-value of 1 × 10^−5^, and the significant enrichment threshold value was padj <0.05 or corrected padj <0.05 for conducting KEGG enrichment analysis. The DEGs were then used to perform co-expression network analysis using the R package WGCNA (Langfelder and Horvath, 2008). All raw sequencing data were deposited at the BioProject under accession code PRJNA944431.

### Quantitative real-time PCR (qRT-PCR) analysis

To validate the accuracy of the RNA-seq data, ten differentially expressed genes were randomly selected to perform qRT-PCR, with β-actin as the internal reference gene. The qRT-PCR verifications were conducted as previously described using a Thunderbird SYBR qPCR Mix (Toyobo, Shanghai, China) and LightCycler480II, 384 (Roche). Fragments were 80–150 bp in length. All primers are listed in Supplementary Table S1. Primer Premier 5.0 application software was used to design RT-qPCR primers, which were synthesized by Bioengineering (Shanghai). The gene level was calculated by using the 2^-∆∆Ct^ method.

### Data analysis

All data were presented as means with standard deviation (SD). The data were analyzed using SPSS 17.0 by one-way analysis of variance (ANOVA). Significance statistical analysis was calculated by Duncan’s multiple range test. *p* < 0.05 indicates a significant difference.

## Results

### Flow cytometry ploidy identification results

**FIGURE 2:**
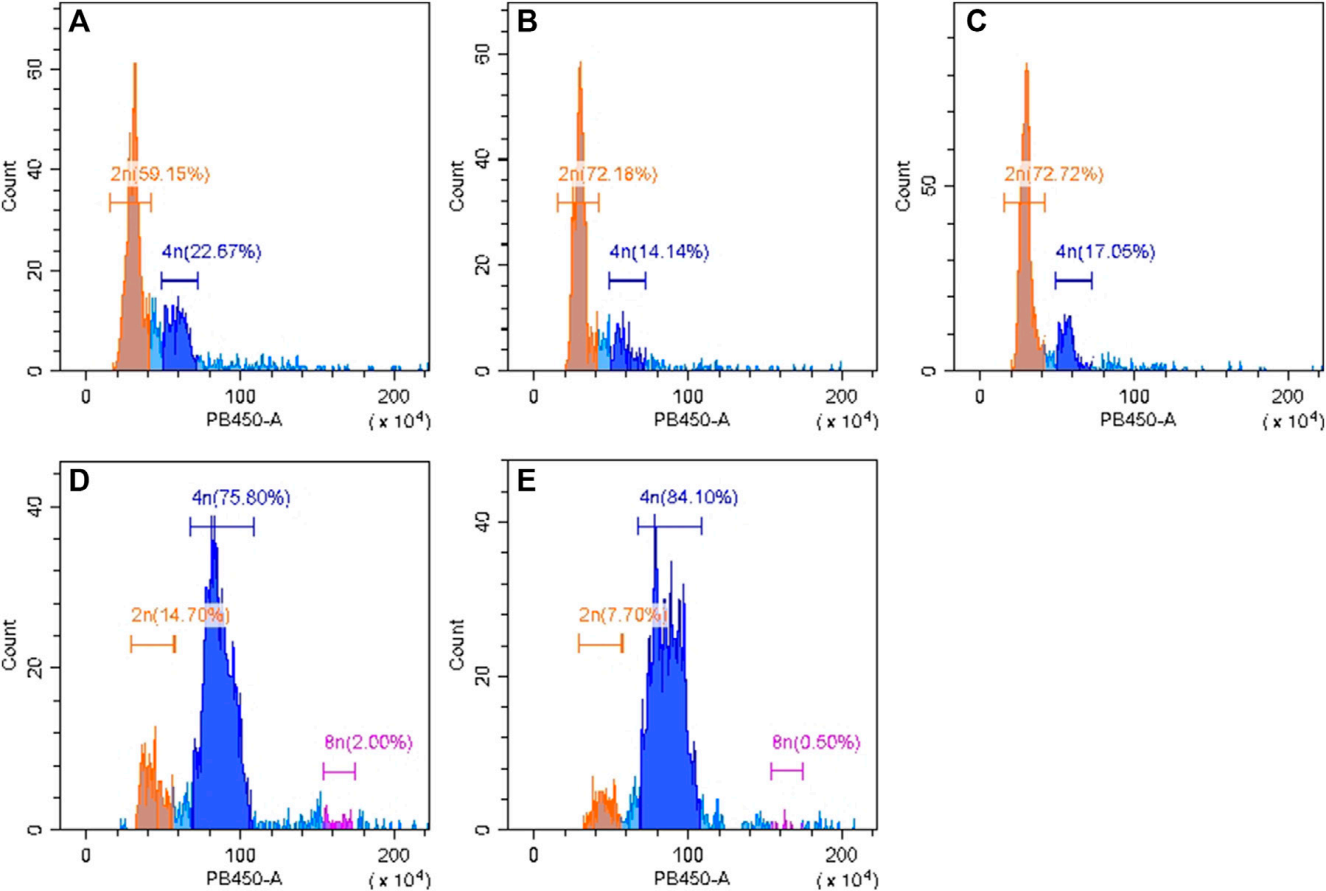
Identification results of flow cytometry analysis for diploid Chinese cabbage Jibai 4 **(A)**, diploid overlapping type cabbage **(B)**, diploid outward-curling type cabbage **(C)**, tetraploid overlapping type cabbage **(D)**, and tetraploid outward-curling type cabbage **(E)**.

Flow cytometry was used to identify chromosome ploidy for the four cultivars (diploid overlapping type cabbage, diploid outward-curling type cabbage, tetraploid overlapping type cabbage, and tetraploid outward-curling type cabbage). The diploid Chinese cabbage Jibai 4 was used as the control group. The results showed that there was only one main peak in Jibai 4. The fluorescence intensity corresponding to the peak was at the position of 50, and the 2n DNA content was 59.15% (Figure 2A). The fluorescence intensity corresponding to the peak value of diploid overlapping type and outward-curling type cabbage was at the position of 60, and the DNA contents of 2n were 72.18% and 72.72%, respectively (Figures 2B, C). The fluorescence intensity corresponding to the peak value of tetraploid overlapping type and outward-curling type cabbage was at the position of 40, and the DNA contents of 4n were 75.80% and 84.10%, respectively (Figures 2D, E). The diploid Chinese cabbage was identified from tetraploid Chinese cabbage using flow cytometry, which further confirmed the accuracy of the test materials. The primer sequences are listed in Table 1.

**TABLE 1:** Primers used in qRT-PCR.

Gene name	Primer sequence (5′ to 3′)	Fragment size/bp
BraA02g030240-F	CAC​CAA​CCC​GAG​CGA​TAA​AG	148bp
BraA02g030240-R	CGG​CTG​CTG​TTG​TTT​CTC​TT	
BraA04g027160-F	CCC​GCA​AGC​TTA​CAA​ACA​CT	154bp
BraA04g027160-R	GAC​ATG​CCT​CTC​GTC​GTC​TA	
BraA02g033740-F	GCG​AAG​GGG​AAG​CAT​TAC​AG	163bp
BraA02g033740-R	CAC​GCA​TCC​TAA​AAG​CAG​CT	
BraA01g042940-F	TCC​GGA​AGA​ACG​TCA​TGT​CA	141bp
BraA01g042940-R	CGT​CCA​TGC​TAA​CCT​TCA​CG	
BraA03g045150-F	AAG​TCC​TGC​CCC​TCG​TTT​GAA	149bp
BraA03g045150-R	CAT​CTG​CCA​TCT​TGC​CAT​CAT	
BraA07g026840-F	CAC​TGC​TTC​TTC​CTC​TGT​TAT​T	151bp
BraA07g026840-R	CGT​TTC​ATC​TTA​TCA​TGA​TTC​C	
BraA09g022320-F	AGT​GAC​GGG​GTT​TAG​CAT​CA	154bp
BraA09g022320-R	ATC​CTC​TTC​CGT​GTT​CCC​TG	
BraA09g064990-F	CAA​AGA​CGG​TGA​CTG​GAT​GC	125bp
BraA09g064990-R	ACT​TCT​CCA​TTG​CTC​TCG​GA	
BraA10g011520-F	AAC​GCT​CCT​GTC​CAT​ATC​GT	144bp
BraA10g011520-R	TGG​CAA​CCC​TGA​TCT​CAC​AT	
BraA07g028010-F	AGT​GTT​GGG​AGT​TCT​CTG​GTC	150bp
BraA07g028010-R	ATT​CCT​GAA​GCA​TTA​ACG​TCA	
β-Actin-F	TAT​GTT​GCT​ATC​CAG​GCC​GT	161bp
β-Actin-R	GTA​AGA​TCA​CGC​CCA​GCA​AG	

### Morphological characteristics of four cultivars

**FIGURE 3:**
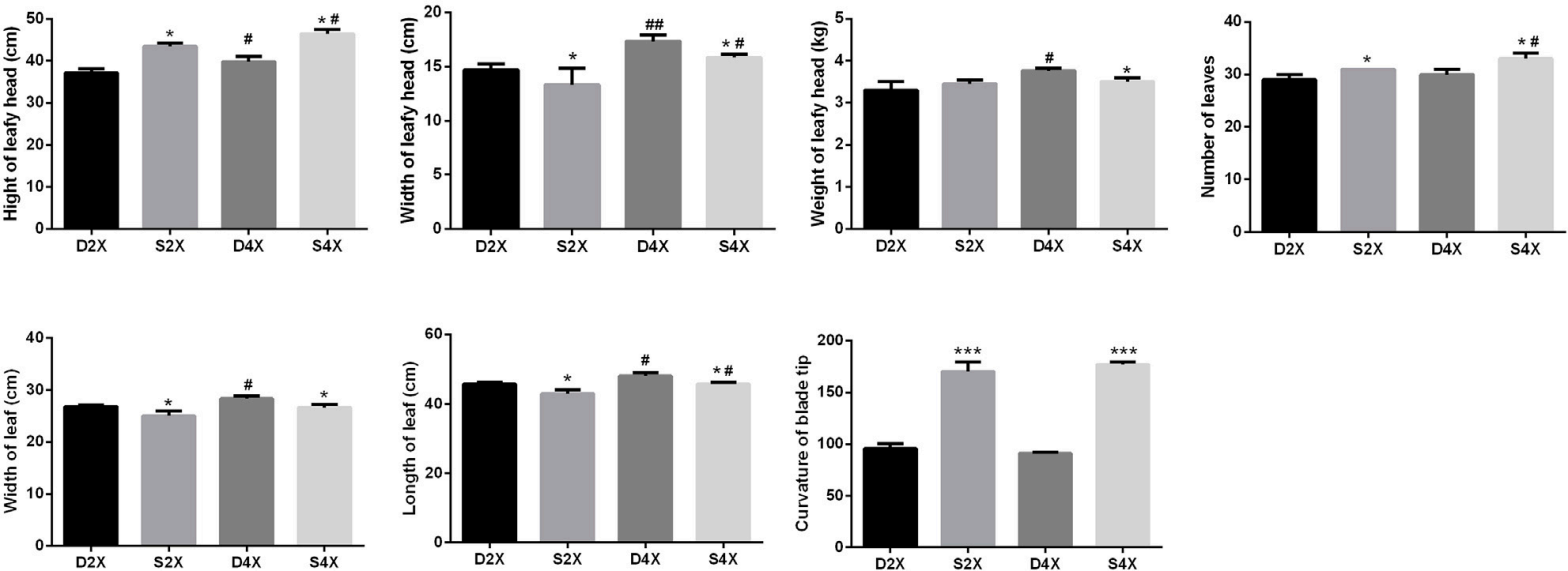
Morphological characteristics of four cultivars, including height, width, and weight of the leafy head, number of leaves, width and length of the leaf, and curvature of the blade tip.

To explore the morphological characteristics of four cultivars, the height, width, and weight of the leafy head, the number of leaves, the leaf width and length, and the curvature of the blade tip were systematically measured (Figure 3). The height of the leafy head, number of leaves, and curvature of the blade tip were evidently higher for the outward-curling type cabbage than those for the overlapping type, while the opposite was true for the width of the leafy head, and leaf width and length within the same ploidy.

In the overlapping type cabbage, the height, width, and weight of the leafy head, and the leaf width and length were higher in the tetraploid than in the diploid plants. The height and width of the leafy head, and the number and length of leaves were more pronounced in the tetraploid than in the diploid outward-curling type cabbage.

### RNA-seq analysis and identification of differentially expressed genes

**FIGURE 4:**
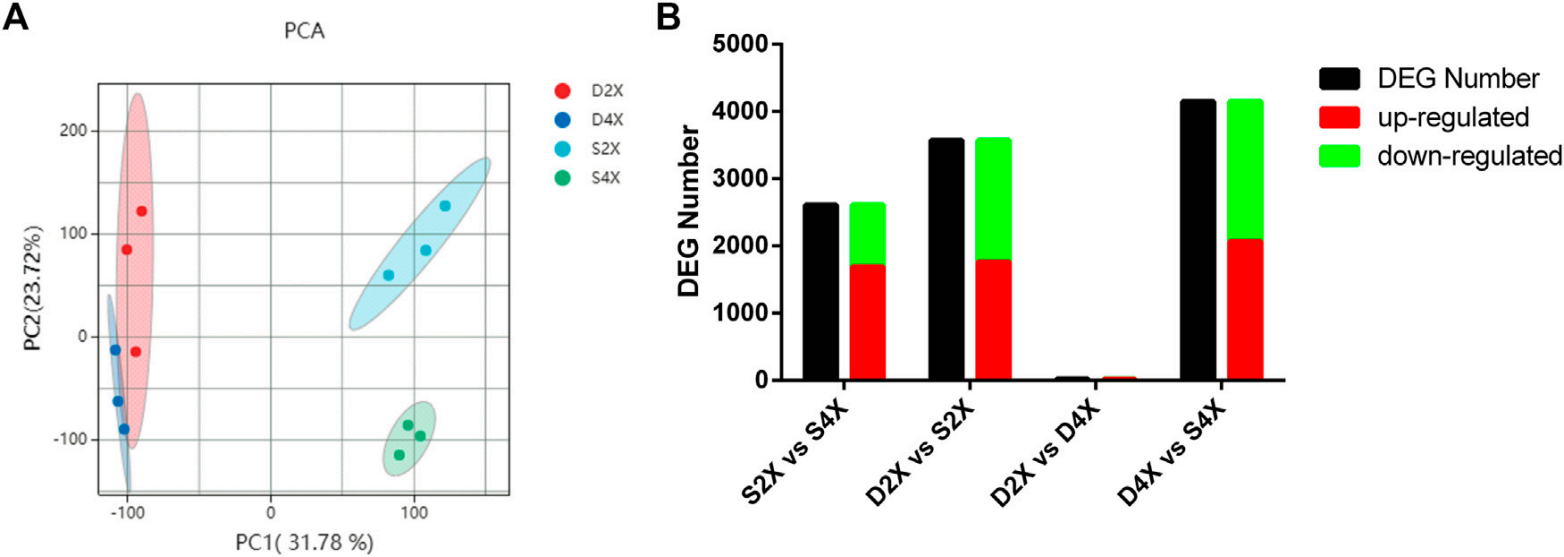
PCA plots of samples **(A)** and significantly up- or downregulated DEG number **(B)**.

Transcriptome analysis obtained 114.66 Gb of clean data at an average of 6.92Gb per sample, with Q30 >95.10%. The clean reads of each sample were sequenced with the reference genome, and the alignment efficiency ranged from 82.35% to 91.77%. Gene expression levels for each replicate were assessed using principal component analysis (PCA), which indicated that the four cultivars had clearly differentiated gene expression (Figure 4A). A total of 2,612 and 3,573 DEGs were obtained in S2X vs S4X and D2X vs S2X (Figure 4B), while 28 and 4,151 DEGs were obtained in D2X vs D4X and D4X vs S4X, respectively (Figure 4B). Detailed information regarding RNA-seq data of each sample is listed in Supplementary Table S5.

**FIGURE 5:**
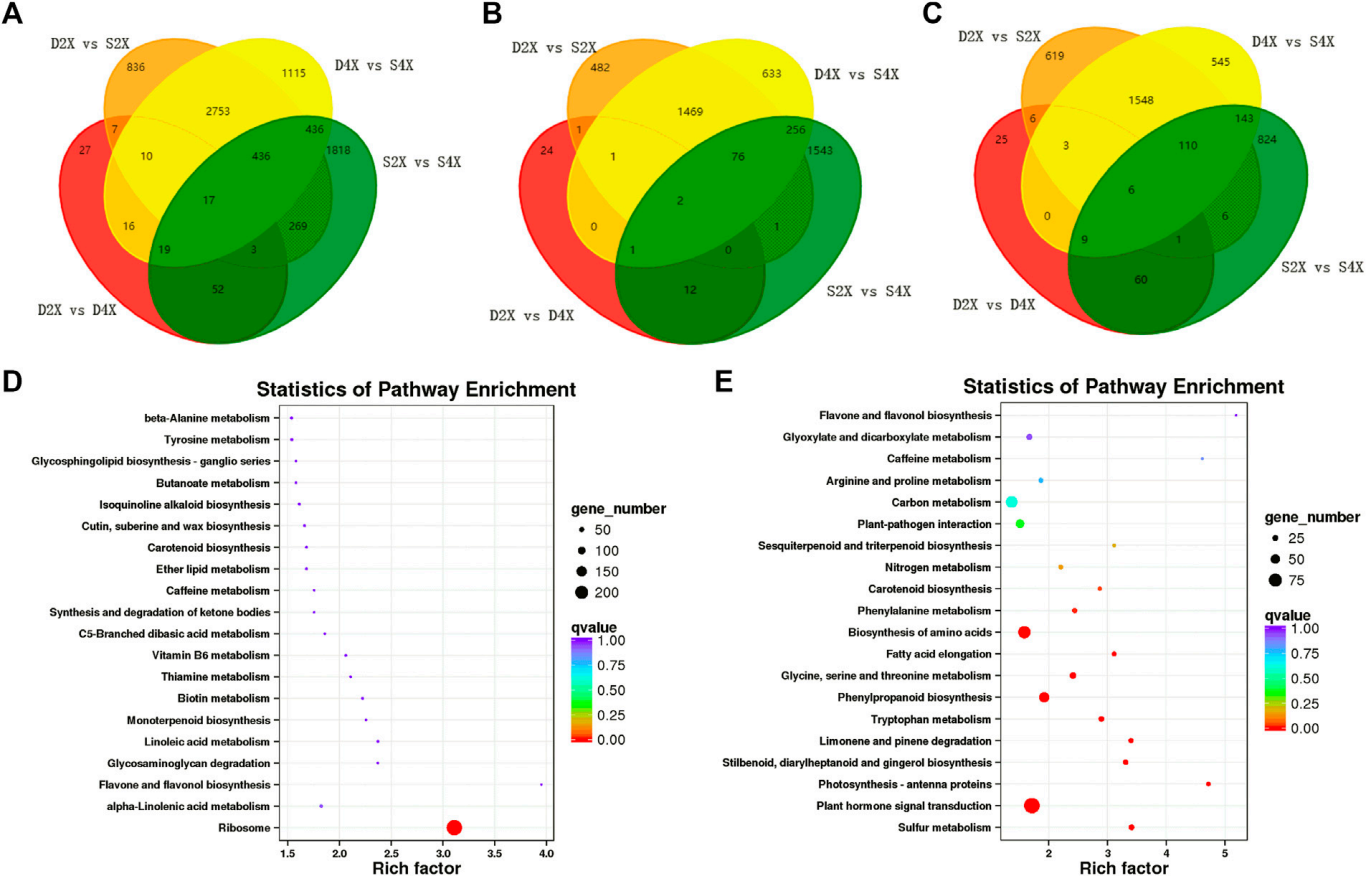
Common and unique DEGs and KEGG enrichment analysis. Venn diagram illustrating common and unique DEGs in different comparison groups **(A)**. Venn diagram of common and unique up- **(B)** and down- **(C)** regulated DEGs in different comparison groups. KEGG pathway enrichment analysis of up- **(D)** and downregulated DEGs **(E)**.

To further identify the genes closely related to the phenotypes of the four varieties, Venn analysis was performed, which displayed unique and common DEGs in each cultivar (Figure 5A). Furthermore, up- and downregulated genes were also exhibited by Venn diagram. Among these DEGs, only two genes were commonly upregulated, while six were downregulated in the four genotypes (Figures 5B, C).

The Venn diagram showed that 24, 482, and 2,176 genes were exclusively upregulated in the D4X, S2X, and S4X genotypes (Figure 5B), while 25, 619, and 1,369 were exclusively downregulated in the D4X, S2X, and S4X genotypes (Figure 5C), respectively. This suggests that the genotype-specific DEGs might contribute to the phenotypic differences.

Subsequently, all DEGs were subjected to KEGG pathway analysis to identify the major metabolic pathways involved. Upregulated genes were significantly enriched in ribosome, alpha-linolenic acid metabolism, starch and sucrose metabolism, and biotin metabolism (Figure 5D). Downregulated genes were mainly enriched in sulfur metabolism, phytohormone signal transduction, photosynthesis-antenna proteins, stilbenoid, diarylheptanoid and gingerol biosynthesis, limonene and pinene degradation, tryptophan metabolism, and phenylpropanoid biosynthesis (Figure 5E).

### Differentially expressed transcription factors in four different genotypes

**FIGURE 6:**
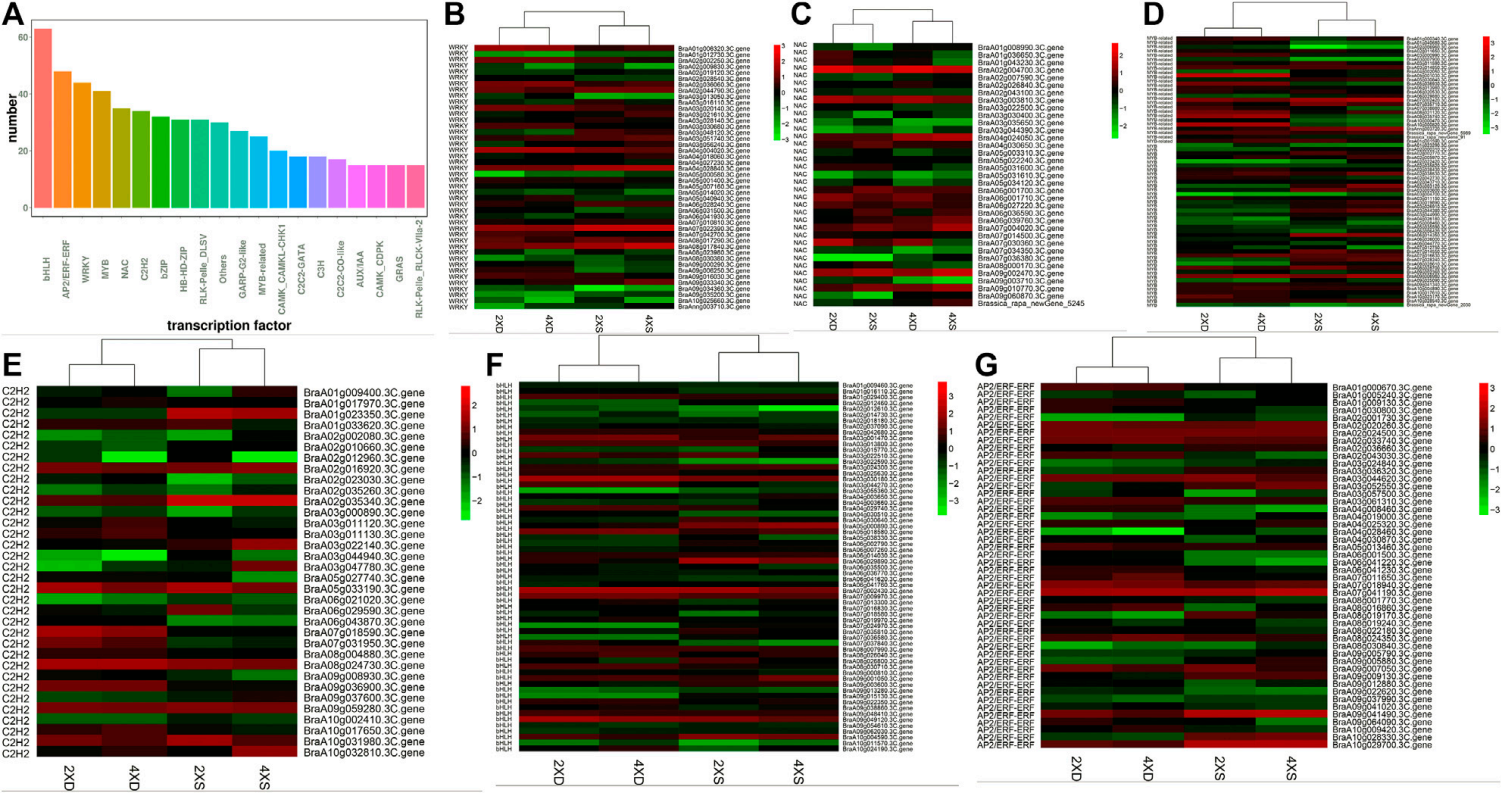
Analysis of TFs in the four cultivars. Bar diagrams show the number of different TF families **(A)**. Cluster analysis of TFs in the four cultivars: WRKY **(B)**, NAC **(C)**, C2H2 **(D)**, MYB **(E)**, bHLH **(F)**, and ERF **(G)**.

Transcription factors (TFs) play a key role in regulating plants’ development and their response to environmental stimuli. The TFs that were identified as DEGs in four different genotypes were highlighted and analyzed. A total of 2,937 TFs were identified, in which the highest rates of TFs belonged to the bHLH, AP2/ERF-ERF, WRKY, MYB, NAC, and C2CH2 families (Figure 6A). Of the 44 identified WRKY DEGs (Figure 6B), *BraA04g004020.3C.*gene was significantly downregulated only in 4XS, while *BraA07g022390.3C*. gene, *BraA08g017840.3C.* gene, and *BraA04g028840.3C*. gene were significantly upregulated in 2XS and 4XS. For 36 differentially expressed NAC genes (Figure 6C), *BraA02g004700.3C*.gene, *BraA07g030360.3C*.gene, and *BraA03g003810.3C*.gene were highly expressed in 2XD. *BraA09g003710.3C*. gene, *BraA07g034350.3C*. gene, *BraA05g034120.3C*. gene, *BraA03g022500.3C*. gene, and *BraA02g043100.3C*. gene were significantly downregulated in 4XD and 4XS. Some 66 MYB DEGs (Figure 6D) were identified, in which more genes such as *BraA05g001030.3C*. gene, *BraA10g000820.3C*. gene, *BraA08g035740.3C*. gene, *BraA05g036930.3C*. gene, and *BraA08g021120.3C*. gene were highly expressed in 2XD and 4XD. Meanwhile, 35 C2H2 (Figure 6E), 63 bHLH (Figure 6F), and 48 ERF (Figure 6G) were differentially expressed in four cultivars, indicating that TFs play a significant role in the phenotypic differences among the four cultivars.

### Co-expression network analysis of genes in different genotypes with WGCNA

**FIGURE 7:**
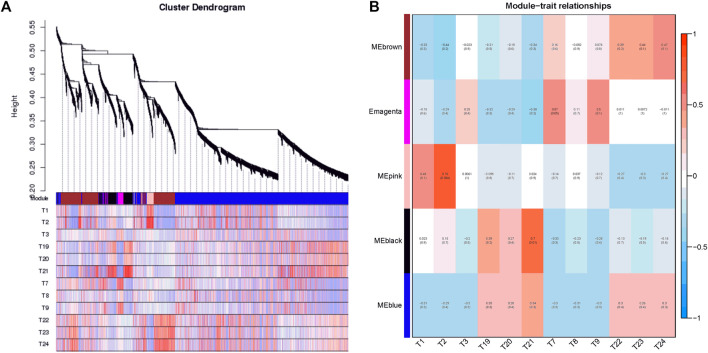
Detection of co-expression network in four cultivars. **(A)** Hierarchical cluster tree showing co-expression modules identified by WGCNA. Each leaf in the tree is one gene. The major tree branches constitute 34 modules labeled by different colors. **(B)** Module–tissue association. Each row corresponds to a module. Each column corresponds to a specific cultivar. The color of each cell at the row–column intersection indicates the correlation coefficient between the module and the cultivar.

In the present study, co-expression networks were built on the basis of pairwise correlations among genes according to the trends of gene expression in all examined samples. The dendogram showed that 34 unique modules were identified, with each module depicted by a different colored branch, and each gene depicted by a leaf (Figure 7A). The gene expression profile of each module was represented by its eigengene—its most notable component. The 34 resulting eigengenes each correlated with unique genotypes due to their genotype-specific expression profiles (Figure 7B).

Notably, five co-expression modules comprised genes that were highly expressed in different cultivars, including MEbrown in tetraploid outward-curling type cabbage (T22, T23, and T24), MEmagenta in tetraploid overlapping type cabbage (T7, T8, and T9), MEpink in diploid overlapping type cabbage (T1, T2, and T3), MEblack in diploid outward-curling type cabbage (T19, T20, and T21), and MEblue in diploid outward-curling type cabbage and tetraploid outward-curling type cabbage. Therefore, each of these five modules identified a specific cultivar or a cluster of genes in two to three similar cultivars. For example, 920 genes involved in the MEbrown module were highly specifically accumulated in tetraploid outward-curling type cabbage, which indicated that this group of genes might be responsible for tetraploid outward-curling head morphotypes. A total of 171 genes involved in MEmagenta were highly specifically accumulated in tetraploid overlapping type cabbage, which indicated that these groups of genes might be responsible for tetraploid overlapping head morphotypes. A total of 195 genes involved in the MEpink module were highly specifically accumulated in diploid overlapping type cabbage, which indicated that this group of genes might be involved in diploid overlapping head morphotypes (Figure 7B). A total of 377 genes involved in MEblack module were highly specifically accumulated in diploid outward-curling type cabbage, which indicated that this group of genes might be involved in diploid outward-curling head morphotypes.

### Phytohormone-related genes may be important participants in phenotypic divergence

**FIGURE 8:**
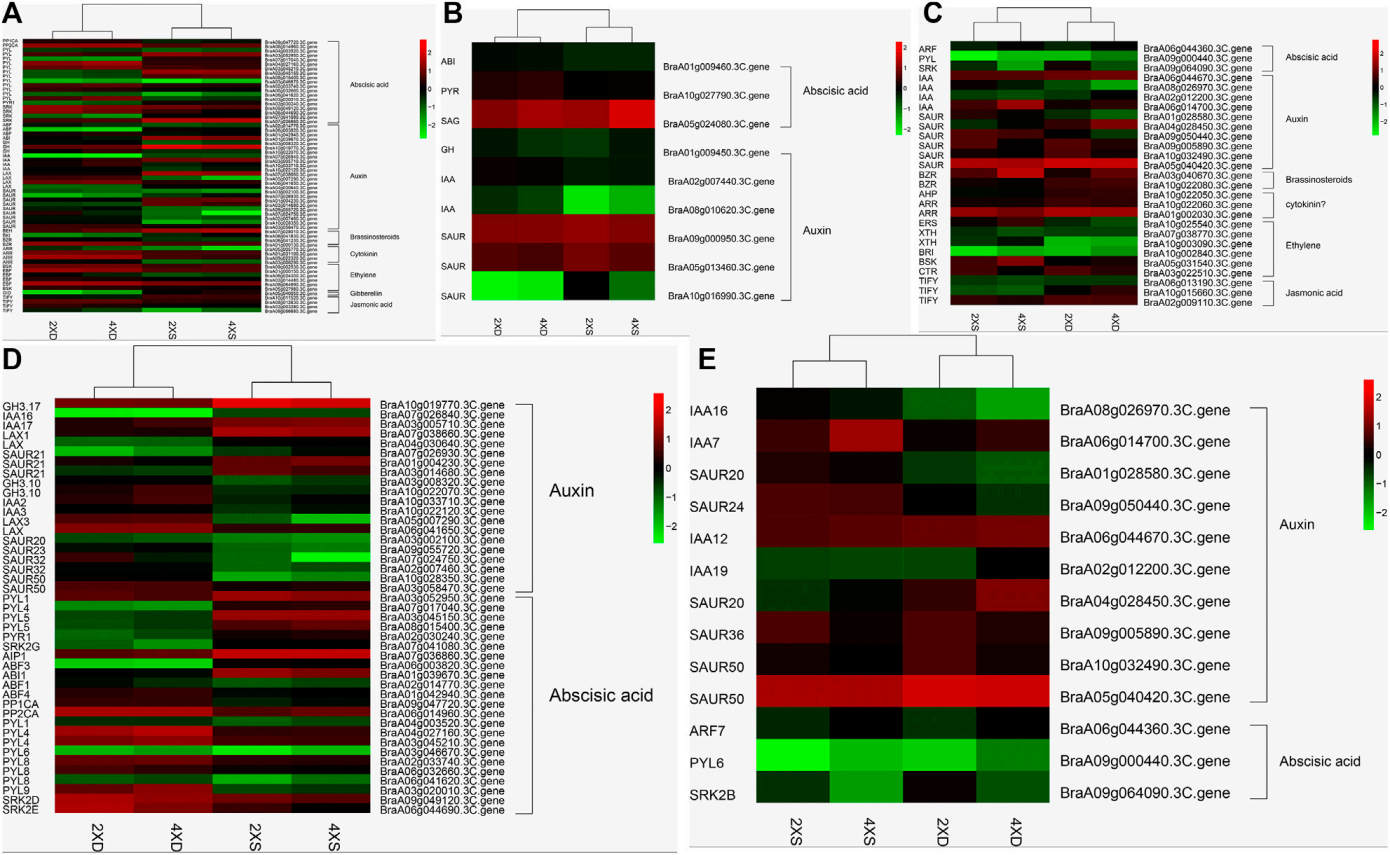
Cluster analysis of 62 identified phytohormone-related genes common to diploid and tetraploid cabbage **(A)** nine DEGs (three abscisic acids and six auxins) unique to diploid cabbage **(B)**, and 27 DEGs unique to tetraploid cabbage **(C)**. Abscisic acid-related genes **(D)**; auxin hormone-related genes **(E)**.

Auxin, acting with other phytohormones, is a key regulator of various developmental processes in plants. Some 113 phytohormone-related DEGs were identified as differentially expressed in four cultivars (Supplementary Table S2). Meanwhile, 62 DEGs common to diploid and tetraploid cabbage (Figure 8A), nine DEGs (three abscisic acids and six auxins) unique to diploid cabbage (Figure 8B), and 27 DEGs unique to tetraploid cabbage were identified (Figure 8C). The DEGs in the auxin hormone pathway, including *GH3.17*, *IAA16*, *IAA17*, *LAX1*, *LAX*, and three *SAUR21*, were evidently highly expressed, while two *GH3.10*, *IAA2*, *IAA3*, *LAX3*, *SAUR20*, *SAUR23*, three *SAUR32*, and two *SAUR50* were lowly expressed in diploid overlapping type cabbage and tetraploid overlapping type cabbage (Figure 8D).

**FIGURE 9:**
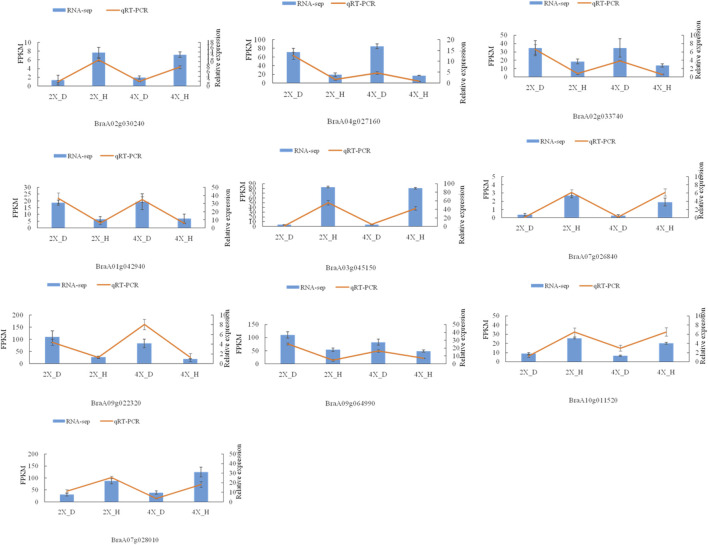
qRT-PCR verification of DEGs. The relative gene expression levels of four cultivars.

Meanwhile, the abscisic acid-related genes *PYL1*, *PYL4,* 2 *PYL5*, *PYR1*, *SRK2G*, *AIP1*, *ABF3*, and *ABI1* were upregulated, while *ABF1*, *ABF4*, *PP1CA*, *PP2CA*, *PYL1*, 2 *PYL4*, *PYL*6, 3 *PYL8*, *PYL9*, *SRK2D*, and *SR K2E* were downregulated in diploid overlapping type cabbage and tetraploid overlapping type cabbage (Figure 8D and Supplementary Table S3). The auxin hormone-related genes *IAA16*, *IAA7*, *SAUR20*, and *SAUR24* were upregulated, while *IAA12*, *IAA19*, *SAUR20*, *SAUR36*, and two *SAUR50* were downregulated in overlapping type cabbage compared to outward-curling type cabbage (Figure 8E; Supplementary Table S4). These results indicated that differential expression for phytohormone-related genes may account for phenotypic divergence. Furthermore, to validate the accuracy of the RNA sequencing, qRT-PCR was conducted to examine the levels of ten genes (Figure 9). The results of qRT-PCR accorded well with the expression data obtained by RNA-seq.

## Discussion

2n gametogenesis is common in plants and plays a very important role in plant sexual polyploidy. 2n gametes can be used in plant polyploid breeding. At present, 2n gametes have been extensively studied, such as in Chinese cabbage (Zhang et al., 2009), potato (Peloquin et al., 2008), kiwi (Seal et al., 2012), sorghum (Hodnett et al., 2019), Arabidopsis (Yi et al., 2023), eggplant (Carputo, 2003), and carnation (Zhou et al., 2015; Zhou et al., 2022). 2n gametes can be utilized to produce new polyploid resources. The common diploid Chinese cabbage, which can produce 2n gametes, and the tetraploid Chinese cabbage material obtained by induction, and the highly fertile tetraploid Chinese cabbage obtained by hybridization were utilized as the material of this study. Phenotypic divergence including the height, width, and weight of the leafy head, the number of leaves, the width and length of leaf, and the curvature of the blade tip were systematically explored. Transcriptome data for these four phenotypes were compared.

TFs are important constituents of plant signaling pathways that define plant development and environmental adaptability, besides playing a role in response to internal signals, and thus coordinate different interacting partners during developmental processes (Xie, 2019; Wani et al., 2021). Previous studies indicated that bHLH/HLH proteins participate in phytohormone signaling and organ development (Dai et al., 2016), which play substantial roles in plant cell elongation. Overexpression of two bHLH (LP1 and LP2) in Arabidopsis individually led to longitudinal polar cell elongation, but single bHLH (LP1 or LP2) exhibited no distinctly altered phenotypes, while double over-expression showed obviously altered phenotypes. Other bHLH/HLH proteins were reported to regulate cell elongation, including positive and negative regulators (Lu et al., 2021). We found that 63 bHLH had evident differential expression in the four cultivars, which, to a certain extent, explained the phenotypic differences. It has been reported that the MYB family directly regulates the development of lateral meristem in Arabidopsis and tomato (Han et al., 2018). Our study showed that many MYB genes were highly expressed in 2XD and 4XD. One of the WRKY transcription factors, *OsWRKY21* (*LOC_Os01g60640*), has been reported to be involved in rice growth and development by regulating the expression of GA metabolism and cell wall biosynthesis-related genes. Overexpression of *OsWRKY21* in rice exhibited a semi-dwarf phenotype, earlier heading dates, and shorter stem internodes (Wei, 2021). As with previous studies, *BraA07g022390.3C. gene*, *BraA08g017840.3C. gene*, and *BraA04g028840.3C.* gene were specifically highly expressed in 2XS and 4XS. *BraA04g004020.3C.* gene showed significantly lower expression in 4XS. *OsDREB2B*, a member of ERF family, negatively regulated plant height in rice, and *OsDREB2B*-overexpressing (OE) rice exhibited dwarf phenotypes, such as reduction in plant height, internode length, and seed length, as well as grain yield (Ma et al., 2022). These reports indicated that these TFs were closely related to the phenotypic divergence of plants. In our research, differential expression of many TFs, including bHLH, AP2/ERF-ERF, WRKY, MYB, NAC, and C2CH2 families, may play important roles in the morphological differences of the four cultivars, such as the height, width, and weight of the leafy head, and the number of leaves. More DETFs suggest the role of a more complex transcriptional regulation network and affected the generation of specific and differential traits.

Plant growth and development, such as cell division, bud development, shoot branching, and senescence, are highly related to plant hormones, resulting in a distinct plant architecture (Zhang, 2020). He et al. (2000) transferred *AUX1* and *AUX2*, two key genes related to auxin in the biosynthesis pathway, into Chinese cabbage. The observation of transgenic Chinese cabbage plants indicated that the high expression of growth hormone-related genes can promote Chinese cabbage entering the pericardium stage ahead of time, increase the number of bulbous leaves, and enhance their weight, thus indicating that auxin regulated the formation of bulbous Chinese cabbage. The formation of leaf bulb type in Chinese cabbage was mainly caused by the bending of the leaves at the top of the bulb. Studies indicate that leaf development is inseparable from the role of phytohormones, in which auxin plays a decisive role in leaf morphological development (Barkoulas et al., 2008). Among the DEGs, 113 phytohormone-related DEGs were identified, and the genes were specifically expressed in different cultivars. Auxin controls leaf development through homeostasis regulation in plants. For example, overexpression of the Arabidopsis auxin homeostasis regulation gene *UGT84B1* changed the balance of auxin content in plants and then caused leaf bending (Jackson et al., 2002). The AXR3 mutant of the auxin gene in *Arabidopsis thaliana* demonstrated the form of an upward curl of leaves (Zgurski et al., 2005). In our study, many of auxin-related genes, including *GH3.17*, *IAA16*, *IAA17*, *LAX1*, and *SAUR21*, were upregulated in diploid overlapping type and tetraploid overlapping type cabbage. Meanwhile, some genes, including *GH3.10*, *IAA2*, *IAA3*, *LAX3*, *SAUR20*, and *SAUR23*, were highly expressed in diploid outward-curling type cabbage and tetraploid outward-curling type cabbage. These results indicate that auxin-related genes were closely related to Chinese cabbage heading and the formation of Chinese cabbage leaf bulb type. IAA regulates the curvature of the top of Chinese cabbage leaves through its own concentration changes. Different IAA content in leaves results in different curvatures, thus forming different types of leaf balls. Our research results identified four kinds of homologous differentially expressed genes (*IAA*, *LAX*, *SAUR*, and *GH3*) in the IAA hormone pathway among the differentially expressed genes of diploid and tetraploid Chinese cabbage leaf bulb types. The interaction between phytohormones controls the growth and development of plants (Xia et al., 2015). Abscisic acid (ABA) is a kind of phytohormone synthesized in plants and plays an important role in seed germination, seedling growth, plant development regulation, stomatal behavior, leaf senescence, abiotic stress, and response to diseases and pests (Cutler et al., 2010; Gomez-Cadenas, 2015). Research shows that, in response to abiotic stress, plants can rapidly synthesize the stress hormone ABA, which stimulates the expression of ABA-induced genes, leads to stomatal closure, reduces transpiration and water loss, and ultimately inhibits cell growth, thereby altering leaf morphology (Peleg and Blumwald, 2011). Many ABA-related genes contribute to the completion of germination and strengthen the idea that cell-wall loosening and remodeling in relation to cell expansion in the embryo axis is a determinant feature in germination (Gimeno-Gilles et al., 2009). ABA-related genes affect stomatal closure and density, and the palisade and spongy tissue distribution of plant leaves (Zhu et al., 2016), which may affect the curvature of leaves and thus affect the type of leaf bulb of Chinese cabbage. In this study, there were six types of homologous genes—*ABF*, *ABI*, *PYL*, *SRK*, *AIP*, and *PP1CA*—in the ABA pathway among the differentially expressed genes shared by diploids and tetraploids, further demonstrating that ABA-related genes play a key role in the formation of leaf bulb types.

In conclusion, comparative-transcriptome analysis can provide insights into key candidate genes related to the formation and phenotypic divergence mechanism of the leafy head. This study indicated that the phytohormone-related genes *IAA7*, *IAA12*, *IAA16*, *IAA19*, *SAUR20*, *SAUR24*, *SAUR36*, *SAUR50 ARF*, *PYL*, and *SRK2B* play an important role in phenotypic difference, especially for tetraploid overlapping type and outward-curling type Chinese cabbage. Furthermore, differently expressed TF genes were selectively analyzed, and seem to be important participants in the phenotypic divergence of head type in Chinese cabbage. These data provide a foundation for elucidating the molecular networks underlying head type in Chinese cabbage.

## Data Availability

The datasets presented in this study can be found in online repositories. The names of the repository/repositories and accession number(s) can be found at https://www.ncbi.nlm.nih.gov/, PRJNA944431.
