# Supplementary material for: Comparative transcriptome analysis provides insights into molecular pathway and genes associated with head-type formation and phenotypic divergence in Chinese cabbage
Source: Front Genet. 2023 May 9;14:1190752. doi: 10.3389/fgene.2023.1190752 (PMC10203174; doi:10.3389/fgene.2023.1190752)
Supplement: Supplementary file 3 [file Table1.DOCX]

|  | Table 1 Primers used in qRT-PCR |  |
| --- | --- | --- |
| **Gene name** | Primer sequence(5'to3') | **Fragment size /bp** |
|  |  |  |
| BraA02g030240-F | CACCAACCCGAGCGATAAAG | 148bp |
| BraA02g030240-R | CGGCTGCTGTTGTTTCTCTT |  |
| BraA04g027160-F | CCCGCAAGCTTACAAACACT | 154bp |
| BraA04g027160-R | GACATGCCTCTCGTCGTCTA |  |
| BraA02g033740-F | GCGAAGGGGAAGCATTACAG | 163bp |
| BraA02g033740-R | CACGCATCCTAAAAGCAGCT |  |
| BraA01g042940-F | TCCGGAAGAACGTCATGTCA | 141bp |
| BraA01g042940-R | CGTCCATGCTAACCTTCACG |  |
| BraA03g045150-F | AAGTCCTGCCCCTCGTTTGAA | 149bp |
| BraA03g045150-R | CATCTGCCATCTTGCCATCAT |  |
| BraA07g026840-F | CACTGCTTCTTCCTCTGTTATT | 151bp |
| BraA07g026840-R | CGTTTCATCTTATCATGATTCC |  |
| BraA09g022320-F | AGTGACGGGGTTTAGCATCA | 154bp |
| BraA09g022320-R | ATCCTCTTCCGTGTTCCCTG |  |
| BraA09g064990-F | CAAAGACGGTGACTGGATGC | 125bp |
| BraA09g064990-R | ACTTCTCCATTGCTCTCGGA |  |
| BraA10g011520-F | AACGCTCCTGTCCATATCGT | 144bp |
| BraA10g011520-R | TGGCAACCCTGATCTCACAT |  |
| BraA07g028010-F | AGTGTTGGGAGTTCTCTGGTC | 150bp |
| BraA07g028010-R | ATTCCTGAAGCATTAACGTCA |  |
| actin-F | TATGTTGCTATCCAGGCCGT | 161bp |
| actin-R | GTAAGATCACGCCCAGCAAG |  |
|  |  |  |
